# Nonunion of Hoffa Fracture Successfully Treated by Total Knee Arthroplasty: A Case Report

**DOI:** 10.7759/cureus.35780

**Published:** 2023-03-05

**Authors:** Toshiki Kitamura, Tomoaki Fukui, Tomoyuki Matsumoto, Keisuke Oe, Kenichi Sawauchi, Ryosuke Kuroda, Takahiro Niikura

**Affiliations:** 1 Department of Orthopaedic Surgery, Kobe University Graduate School of Medicine, Kobe, JPN

**Keywords:** open reduction and internal fixation (orif), nonunion fracture, total knee replacement (tkr), nonunion of distal femur, hoffa’s fracture

## Abstract

A Hoffa fracture is a rare type of femoral fracture that is difficult to treat. Nonoperative treatments usually result in failure; hence, in most cases, surgical treatments are essential. Nonunion following Hoffa fracture appears to be relatively uncommon, and there are limited reports in the literature about this type of nonunion. These reports suggest that open reduction and rigid internal fixation is the standard treatment for this type of nonunion.

This study reports the case of a 61-year-old male patient who suffered from left lateral Hoffa fracture after falling from a truck bed. At the former hospital, open reduction and internal fixation were performed with plates and screws at 8 days post-injury. Postoperatively, displacement of the lateral proximal fragment was observed, and the patient reported left knee pain. Therefore, a revision open reduction and internal fixation was performed 4 months post-surgery. However, 6 months after the revision surgery, the patient reported instability and pain in the left knee, and subsequent radiography revealed nonunion of the fracture in the lateral condyle.

The patient was referred to our hospital for further treatment. Treatment by re-revision open reduction and internal fixation was deemed challenging, and so rotating hinge knee (RHK) arthroplasty was performed as a salvage treatment. At 3 years post-surgery, no significant problems were observed, and the patient could walk without any assistance. The range of motion of the left knee was 0 to 100° without extension lag, and there was no lateral instability.

Standard treatment for Hoffa fracture nonunion is commonly anatomical reduction and rigid internal fixation. However, total knee arthroplasty may be a better option for the treatment of Hoffa fracture nonunion in older patients.

## Introduction

Hoffa fractures are intra-articular coronal plane fractures of the femoral condyles and occur in the lateral condyle more commonly than in the medial condyle [1]. Fractures of this type are difficult to treat, and nonoperative treatment usually results in poor outcomes [2]. Accurate anatomical reduction and rigid internal fixation are required to achieve successful results [3]. Currently, while some reports demonstrate that surgical treatments of Hoffa fractures have positive outcomes, the surgical approach and fixation method are still controversial [4-6]. Nonunion and mal-union are common complications with this type of fracture; however, there are few reports concerning salvage treatment for nonunion of Hoffa fracture, in which open reduction and internal fixation (ORIF) with or without bone grafting was mainly performed [6,7].

This study reports the case of a patient with nonunion of Hoffa fracture, successfully treated by total knee arthroplasty (TKA).

## Case presentation

**Figure 1:**
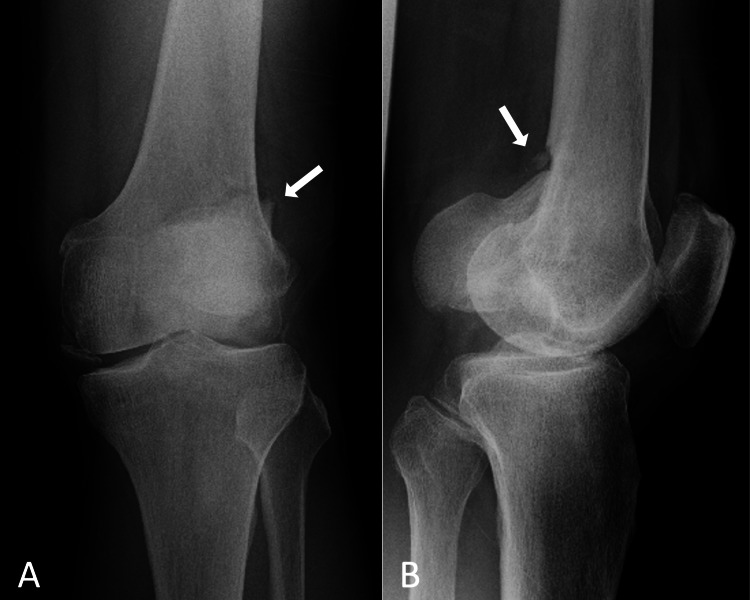
Radiographs of the left knee taken after the injury. A: frontal radiograph, B: lateral radiograph Lateral Hoffa fracture with displacement and articular surface comminution are observed. Fracture lines are shown by arrows.

**Figure 2:**
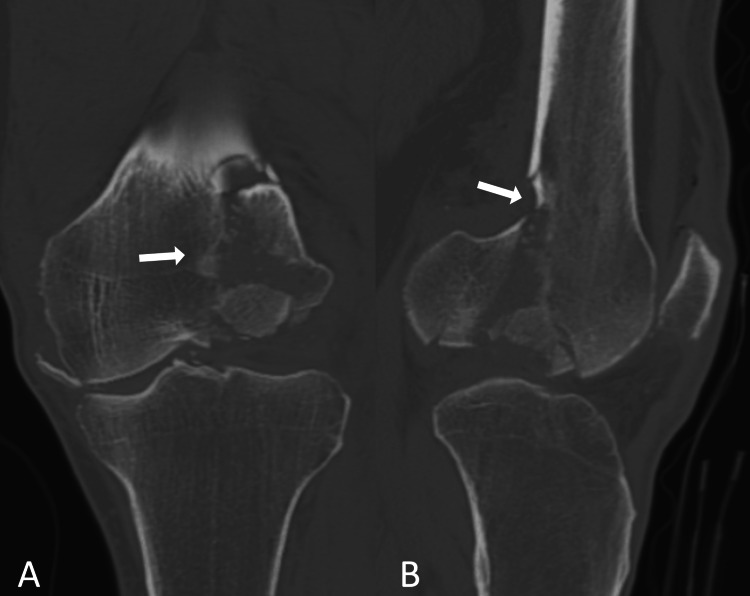
Coronal and sagittal views of the left knee via CT scans. A: coronal view of CT scans, B: sagittal view of CT scans Displacement and comminution of fracture site are shown more clearly than with radiography. Displacement and comminution of the fracture site are shown by arrows.

**Figure 3:**
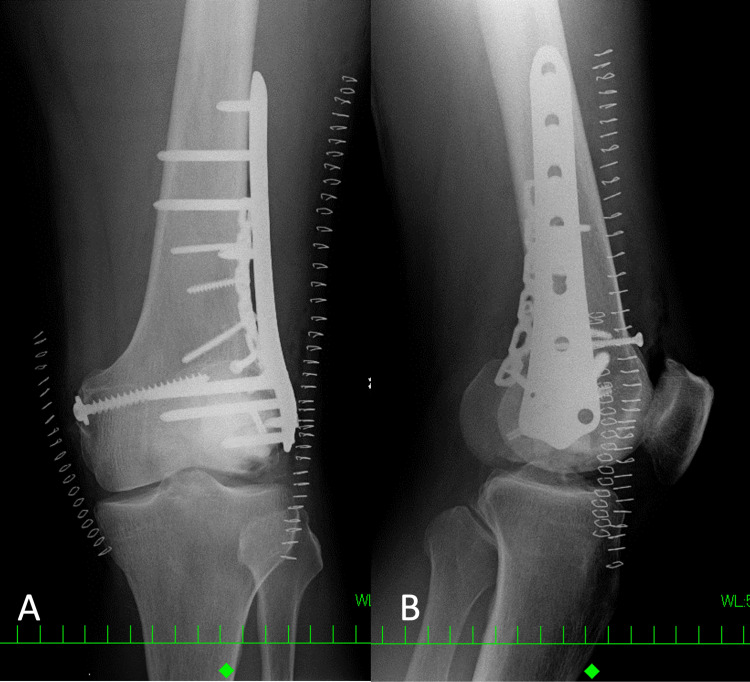
Radiographs taken after the initial ORIF by plates and screws in the former hospital. A: frontal radiograph, B: lateral radiograph ORIF: open reduction and internal fixation The displacement of the lateral posterior fragment looks persistent.

**Figure 4:**
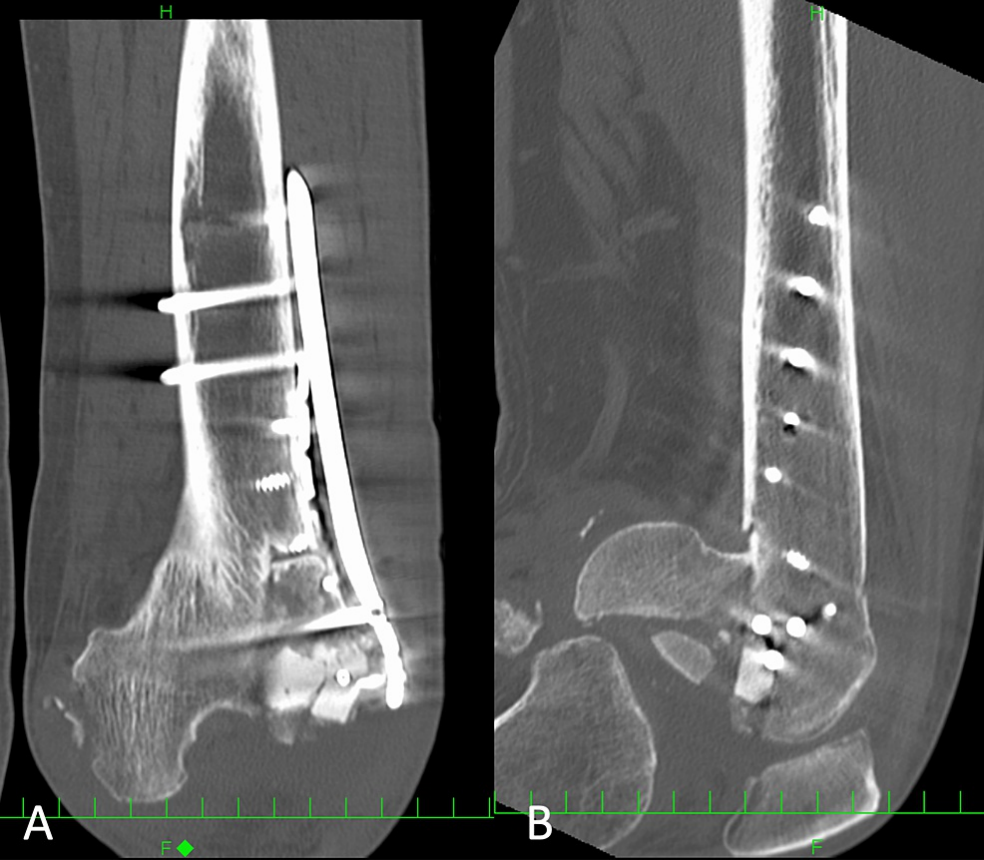
Coronal and sagittal views of the left knee via CT scans after the initial ORIF. A: coronal view of CT scans, B: sagittal view of CT scans ORIF: open reduction and internal fixation Postoperative displacement and comminution can be observed.

**Figure 5:**
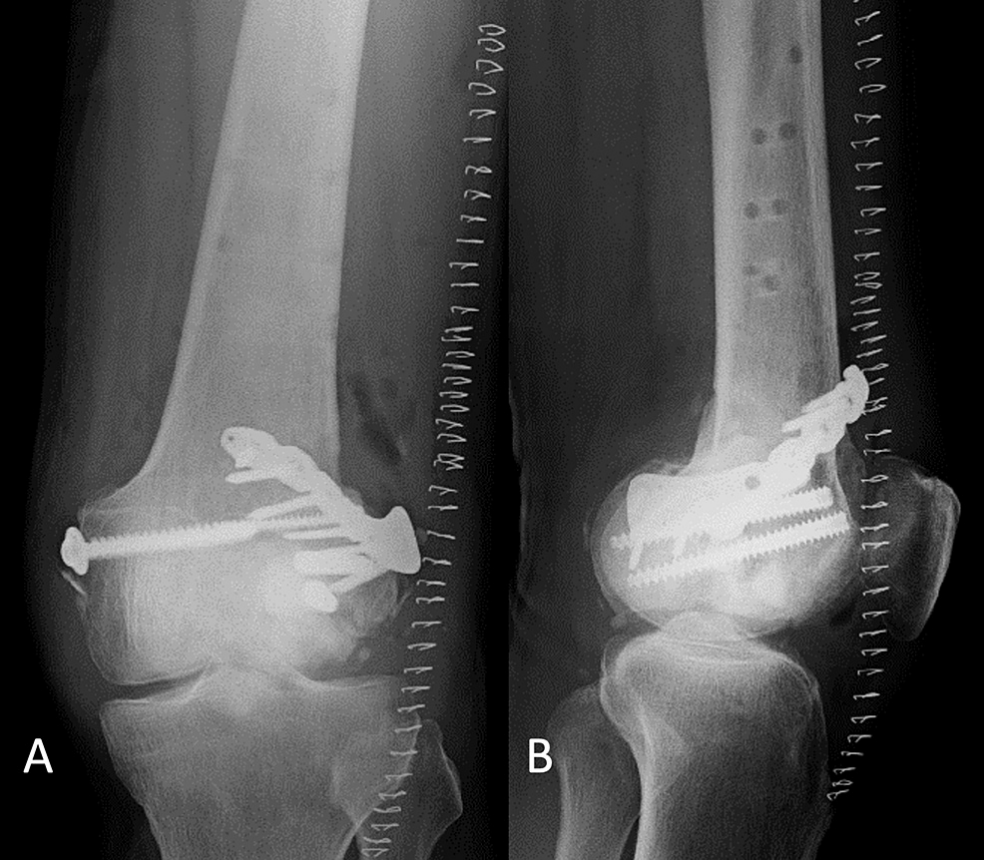
Radiographs taken immediately after revision ORIF in the former hospital. A: frontal radiograph, B: lateral radiograph ORIF: open reduction and internal fixation Two existing plates were exchanged to single clavicle plate. Lateral femoral articular surface appears still irregular.

A 61-year-old male patient fell from a truck bed causing high-energy trauma to the left knee. On radiography and computed tomography (CT) scans, the patient was diagnosed with a left lateral Hoffa fracture with comminution of the articular surface in a former hospital (Figures 1-2). ORIF was performed using two plates and screws, and artificial bone grafting of the bone defect was performed 8 days post-injury at the aforementioned hospital. The bone fragment, to which the medial collateral ligament was attached, was fixed in place with a screw and washer (Figure 3). During postoperative follow-up, the patient reported persistent left knee pain, and CT scans revealed displacement of the lateral proximal fragment (Figure 4). Therefore, revision ORIF, including an exchange of two plates to one locking clavicle plate, was performed approximately 4 months post-initial surgery at the same hospital (Figure 5). Although partial weight bearing was permitted 6 months after the revision ORIF, the patient could not walk owing to instability and pain in the left knee, and the fracture in the lateral condyle was not united on radiographs. 

**Figure 6:**
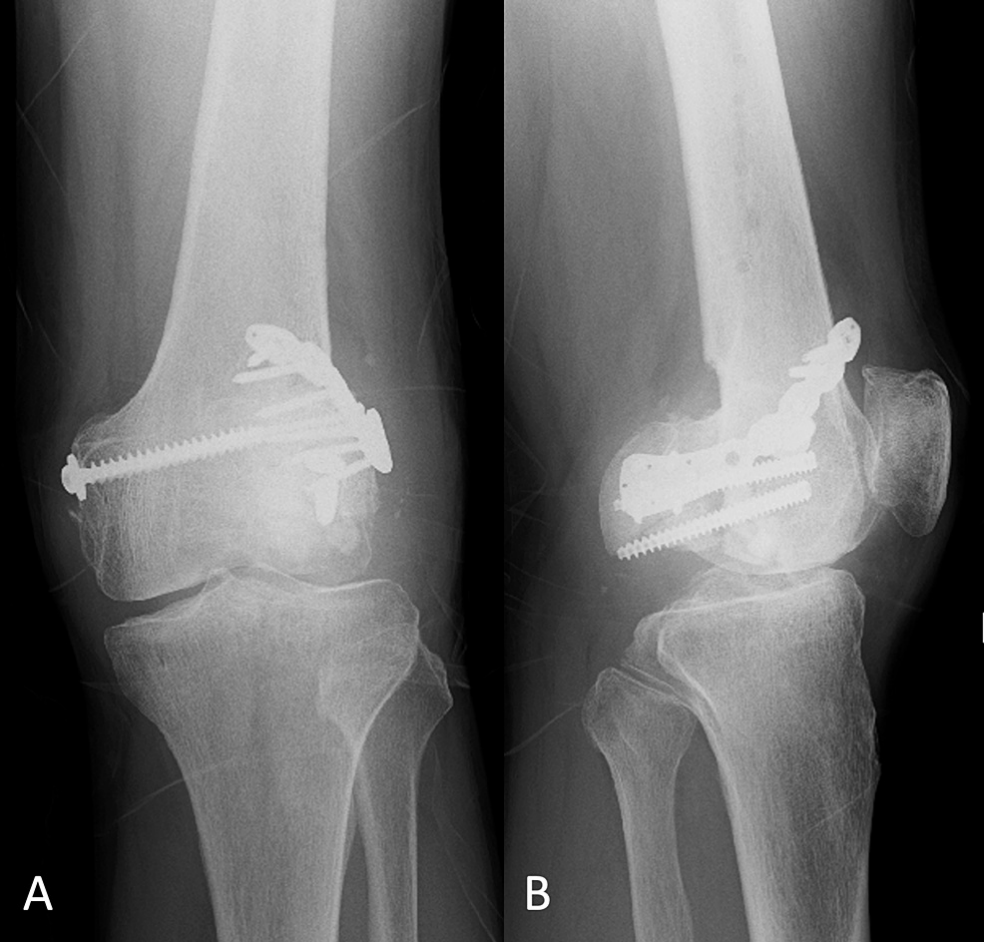
Initial radiographs taken at our hospital. A: frontal radiograph, B:lateral radiograph The fracture site appears ununited in lateral view.

**Figure 7:**
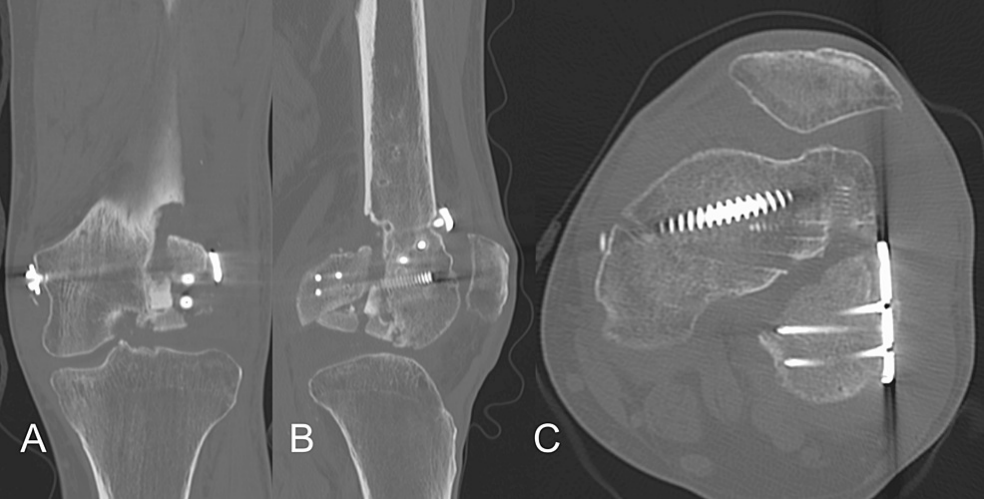
Coronal, sagittal, and axial views of the left knee via first CT scans taken at our hospital. A: coronal view of CT scans, B: sagittal view of CT scans, C: axial view of CT scans Displacement and gap in the nonunion site are demonstrated.

Therefore, the patient was referred to our hospital for further treatment. On physical examination, the left knee displayed showed swelling, joint effusion, lateral medial valgus instability, and tenderness on the lateral side, and the range of motion (ROM) was limited to 0° in extension and 85° in flexion, without extension lag, which induced pain. The left knee pain was induced only in passive and active maximum flexion. The patient could walk with bilateral axillary double crutches but had constant pain on the lateral side of the left knee. Radiographs and CT scans revealed nonunion following the left lateral Hoffa fracture with displacement including the articular surface (Figures 6-7). We confirmed that the culture of aspirate from left knee joint was negative. We performed puncture of the left knee joint was performed, and joint fluid culture was negative. The patient had a previous history of myocardial infarction at 45 years of age and type 1 diabetes mellitus since his late 20s. He had started smoking at 20 years of age and quit smoking at 45 years of age.

**Figure 8:**
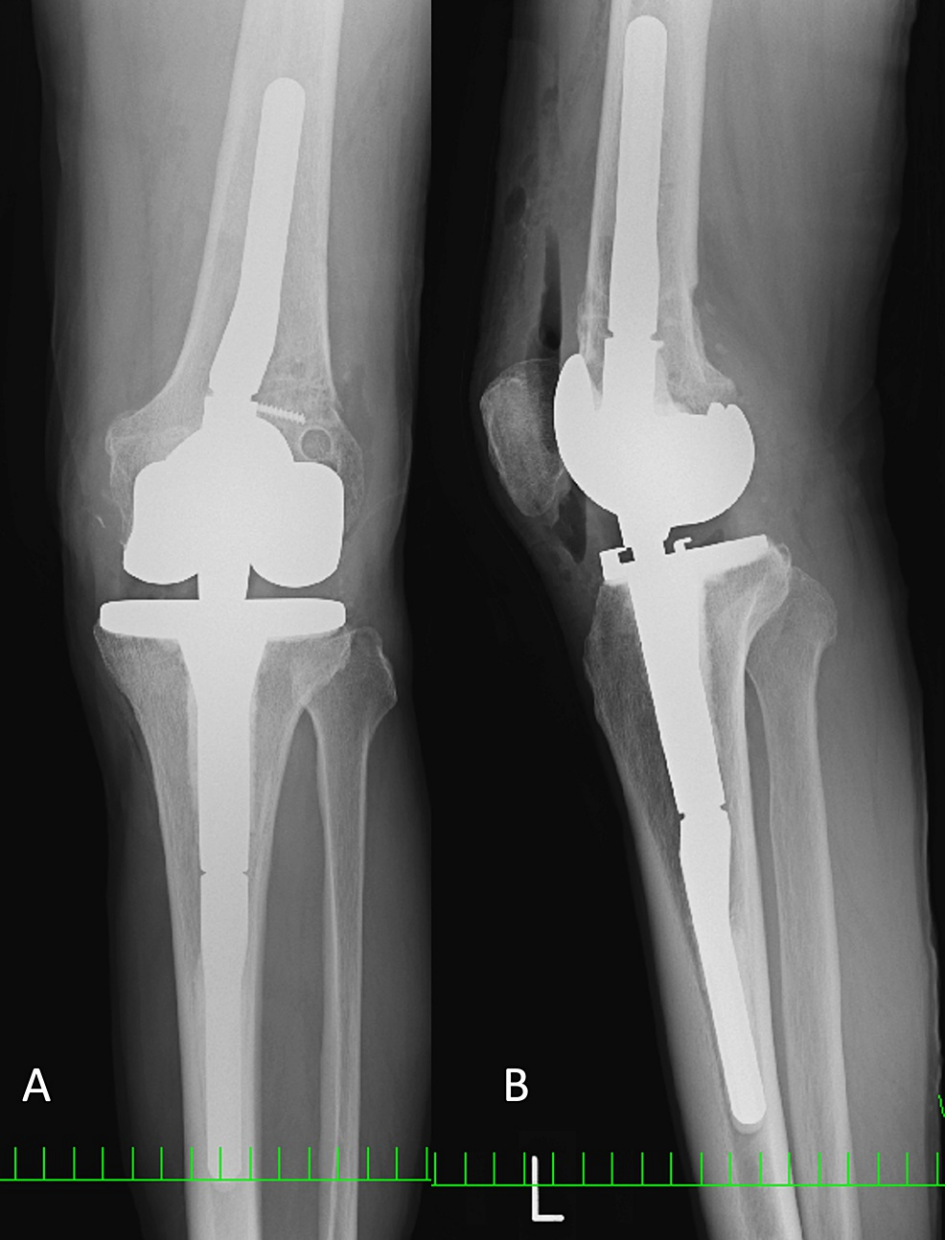
Radiography after TKA. A: frontal radiograph, B: lateral radiograph TKA: total knee arthroplasty; RHK: rotating hinge knee RHK type looks installed appropriately.

It was determined that treatment by re-revision ORIF was not impossible but exceedingly complicated. Considering the patient’s desire to return to work as early as possible, TKA was performed as a salvage treatment (Figure 8). Considering the significant lateral instability of the knee, rotating hinge knee (RHK) (NexGen RHK, Zimmer-Biomet, Warsaw, Indiana, US) arthroplasty was selected for the treatment method. In the operation, we dealt with lateral bone defects by adding augmentation block. Weight-bearing without limitation and ROM exercises were permitted on the next day following surgery, and no significant problems were observed throughout the postoperative course. He was discharged home with a single crutch gait 2 weeks after the operation. On 8 months postoperatively, he became able to return to work. He was discharged to home with single crutch gait 2 weeks after the operation. At 8 months post-surgery, he became able to return to work. At 3 years post-surgery, the patient could walk without assistance and returned to work as an office worker. The ROM of the left knee was 0° to 100° without extension lag, and there was no observed lateral instability. No abnormal findings regarding the implant were identified on radiographs; therefore, the patient was highly satisfied.

## Discussion

Fractures in the coronal planes of the femoral condyles are rare and account for 8.7 to 13% of distal femoral fractures [4,8]. Hoffa first described this type of fracture in 1904 [9]. These fractures can also be classified as type 33-B3 fractures based on the AO classification.

It is well known that treatment for Hoffa fractures is difficult, and nonoperative treatments may cause severe complications, such as nonunion and malunion [2]. There are few reports in the literature concerning nonoperative treatment for Hoffa fractures; however, indications for nonoperative treatment should be carefully considered. Most incidences of Hoffa fractures, even particularly displaced ones, require ORIF to achieve successful results; however, the surgical approach and fixation technique are still controversial [10,11]. Lu et al. reported that fixation with compression screw and buttress plate is effective and reliable [12]. However, the instability of the fracture site, in this case, was supposed too severe to be stabilized with the applied plates and screws, resulting in nonunion.

There are a limited number of reports regarding treatment for nonunion of Hoffa fracture. Nandy et al. described a case of nonunion after Hoffa fracture in a 16-year-old boy, who underwent ORIF with bone grafting and fixation using a lag screw and neutralization plate, resulting in a radiographically and functionally good outcome [13]. McDonough and Bernstein also reported a case of a Hoffa fracture nonunion in a child who was successfully treated using two cancellous screws leading to a good outcome [14]. Similarly, Jiang et al. treated nonunion following a Hoffa fracture in a 27-year-old patient by using two lag screws and a dynamic plate fixation with bone grafting [15]. A study by Strauss et al. described treatment by ORIF with corticocancellous bone screws and two partially threaded cancellous screws [15]. Singh et al. described six cases of nonunion of Hoffa fractures over 3 years. In these reports, all patients were treated using ORIF with screws, and in two cases, using locking reconstruction plates [16].

In the abovementioned reports, all the patients with nonunion of Hoffa fracture were treated using ORIF with or without bone grafting, and good long-term functional results were obtained. All reports suggest that standard treatment for Hoffa fracture nonunion is an accurate anatomical reduction and rigid internal fixation with bone graft, if necessary.

On the other hand, there are two reports about TKA for Hoffa nonunion. Albuquerque et al. advocated TKA as a viable option for such elderly patients with Hoffa nonunion, which supports our study [17]. Reddy et al. reported two cases of bicondylar Hoffa fractures with implant failure and nonunion treated successfully with long-stem TKA [18]. In this report, it is suggested that long-stem TKA might be a good treatment option for complex situations, as in the current case.

As for the current case, osteosynthesis was considered a possibility; however, the procedure is regarded as being particularly challenging and the time taken for bony union formation is lengthy. At the time of referral, the patient had been absent from work for one year due to difficulty in walking and he indicated his desire to return as soon as possible. Considering his age of 61 years, TKA was selected as a salvage treatment for the nonunion.

Today, due to the improvement of implants, age indication for TKA has been expanded. However, the risk of prosthesis failure and aseptic loosening in younger patients remains high [19]. According to a report by Julin et al. regarding the relationship between age and implant survival, the implant 5-year survival rates were 92% in patients aged ≤ 55 years, 95% in patients aged 56-65 years, and 97% in patients who were > 65 years of age [20]. In younger patients, ORIF might be considered as the treatment of choice before TKA. However, TKA was the optimal treatment choice for the 61-year-old patient in this study and it led to a good outcome.

This case report has several limitations such as the lack of generalizability, inability to establish a cause-effect relationship, the danger of overinterpretation, and publication bias. Further studies are required to validate if the current treatment for similar cases is appropriate.

## Conclusions

In conclusion, this study reports the case of a patient who suffered from nonunion following a Hoffa fracture and was treated successfully by TKA. TKA could be regarded as a better option for the treatment of Hoffa fracture nonunion in older patients.
